# Decline in frequency of the 2La chromosomal inversion in *Anopheles gambiae* (*s.s*.) in Western Kenya: correlation with increase in ownership of insecticide-treated bed nets

**DOI:** 10.1186/s13071-016-1621-3

**Published:** 2016-06-10

**Authors:** Damaris Matoke-Muhia, John E. Gimnig, Luna Kamau, Josephat Shililu, M. Nabie Bayoh, Edward D. Walker

**Affiliations:** Centre for Biotechnology Research and Development, Kenya Medical Research Institute, P.O. Box 54840-00200, Nairobi, Kenya; Institute of Tropical of Medicine and Infectious diseases, Jomo Kenyatta University of Agriculture and Technology, P.O. Box 62000-00200, Nairobi, Kenya; Division of Parasitic Diseases and Malaria, Center for Disease Control and Prevention, Atlanta, GA USA; Centers for Disease Control and Prevention, PO Box 1578, Kisumu, Kenya; Department of Microbiology and Molecular Genetics, Michigan State University, East Lansing, MI 48824 USA

**Keywords:** Chromosomal inversion, *Anopheles gambiae*, Insecticide-treated nets

## Abstract

**Background:**

The 2La chromosomal inversion, a genetic polymorphism in *An. gambiae* (*sensu stricto*) (*s.s*.), is associated with adaptation to microclimatic differences in humidity and desiccation resistance and mosquito behaviors. Ownership of insecticide-treated bed nets (ITNs) for malaria control has increased markedly in western Kenya in the last 20 years. An increase in the frequency of ITNs indoors could select against house entering or indoor resting of *Anopheles* mosquitoes. Thus, the frequency of the 2La inversion is postulated to change in *An. gambiae* (*s.s*.) with the increase of ITN ownership over time.

**Methods:**

*Anopheles gambiae* mosquitoes were sampled between 1994 and 2011 using pyrethrum knockdown, bednet traps and human landing catches (HLC) from Asembo and Seme, western Kenya. The 2La inversion was detected by a PCR assay with primers designed for proximal breakpoints of the 2La/a and 2L+^a^/+^a^ chromosomal conformation. Mosquitoes were tested for malaria parasite infection by sporozoite ELISA.

**Results:**

The frequency of the 2La chromosomal inversion declined from 100 % of all chromosomes in 1994 to 17 % in 2005 and remained low through 2011 (21 %). ITN ownership increased from 0 to > 90 % of houses in the study area during this interval. The decline in the frequency of the 2La chromosomal inversion was significantly, negatively correlated with year (*r* = -0.93) and with increase in ITN ownership (*r* = -0.96). The frequency of the homo- and heterokaryotypes departed significantly from Hardy-Weinberg equilibrium, suggesting that 2La/a karyotype was under selection, earlier in its favor and later, against it. Precipitation and maximum monthly temperature did not vary over time, therefore there was no trend in climate that could account for the decline. There was no significant difference in frequency of the 2La inversion in *An. gambiae* (*s.s*.) females sampled indoors or outdoors in HCL in 2011, nor was there an association between the 2La inversion and infection with *Plasmodium falciparum* sporozoites.

**Conclusions:**

The increase in ITN ownership in the study area was negatively correlated with the frequency of 2La inversion. The decline in 2La frequency in western Kenya is postulated to be due to differential impacts of ITNs on mosquitoes with different 2La karyotypes, possibly mediated by differences in behavior associated with the 2La karyotypes. Further research is required to determine if this is a widespread phenomenon, to further determine the association of the 2La karyotypes with mosquito behavior, and to assess whether ITNs are exerting selection mediated by differences in behavior on the different karyotypes.

## Background

Malaria is one of the most important causes of morbidity and mortality in sub-Saharan Africa. The primary interventions against malaria include prompt diagnosis and treatment of human infections with effective medicines; use of intermittent drug therapies in high-risk populations; distribution and use of insecticide-treated nets (ITNs) to achieve high coverage of human populations at risk of malaria; and indoor house spraying of residual insecticides [1]. The three primary species of malaria vectors in sub-Saharan Africa, *Anopheles gambiae* Giles*, Anopheles arabiensis* Patton and *Anopheles funestus* Giles, each have attributes that contribute to the magnitude of their vectorial capacity; in particular, their tendency to feed on humans (anthropophily) and their close association with human dwellings (endophily).

Vector control relies largely on use of synthetic insecticides, delivered either through indoor residual spraying (IRS) or ITNs that are deployed indoors. The sole class of chemicals used as the active ingredient in ITNs is the pyrethroids, which show strong toxicological and behavioral effects on mosquitoes including contact lethality, knockdown and excito-repellency after contact [2–4]. In a study conducted in western Kenya, an equal number of *An. gambiae* (*s.s*.) entered houses with or without permethrin-treated bed nets, but a greater number exited houses with these ITNs compared to houses without them [5]. These observations generally demonstrate that female *An. gambiae* (*s.s*.) readily enter the indoor environment, respond to environmental stressors and stimulants encountered there, and adjust their spatial distribution in response to those stimulants.

The taxon *An. gambiae* (*s.l.*) is remarkably polymorphic, representing a complex of closely related species with distinct biological attributes [6]. It is also a highly variable but “true” species when considering *An. gambiae* (*s.s*.), whose evolution and adaptations to environmental conditions have been molded by chromosomal inversion polymorphisms [6–8]. *Anopheles gambiae *(*s.s*.) is widely distributed throughout sub-Saharan Africa; it is one of the most proficient vectors of malaria on the continent. The other species within the *An. gambiae* (*s.l*.) complex are distinguished generally by fixed chromosomal arrangements [9, 10]. *Anopheles arabiensis* and *An. merus* Dönitz are monomorphic for the 2La/a (inversion) arrangement, whereas *An. bwambae* White, *An. melas* Theobald and *An. quadriannulatus* Theobald are fixed for the alternative arrangement 2L+^a^/+^a^ (the so-called standard arrangement).

*Anopheles gambiae* (*s.s*.) is a predominant member of the *An. gambiae* (*s.l.*) complex in which the 2La chromosome is polymorphic, having 2L+^a^/+^a^ (homokaryotype standard), 2La/+^a^ (heterokaryotype) and 2La/a (homokaryotype inversion) arrangements [9, 10]. These various chromosomal conformations contribute to sub-structuring of *An. gambiae *(*s.s*.) populations [7, 9–12], suggesting incipient speciation [13] and leading recently to the description of *Anopheles coluzzii* Coetzee & Wilkerson, formerly known as the “M form” of *An. gambiae* (*s.s*.) and separating it from the so-called “S form” of *An. gambiae* (*s.s*.) [14].

The sampling distribution of individuals of *An. gambiae* (*s.s*.) with different conformations of the 2La karyotype is associated with particular behaviors and microhabitats in the human living environment [12]. Associative studies suggest that the 2La chromosome inversion contributes to the adaptation of *An. gambiae* (*s.s*.) to arid conditions [6, 9, 15]. Likely, it may have entered *An. gambiae* (*s.s*.) populations some thousands of years ago by genetic introgression from *An. arabiensis*, a species that is homokaryotypically-fixed for 2La and well known to have a wider geographic distribution into hotter, drier areas [16]. Individuals with the wild type karyotype (2L+^a^/+^a^) are associated with more humid climates, whilst individuals with the inverted conformation (2La/a) are more common in dry climates [15]. The frequency of the 2La inversion within an *An. gambiae* (*s.s*.) population changes in response to seasonal fluctuations in rainfall, demonstrating that as a genotype it is sensitive to local environmental processes [7, 11, 15]. Survival of adult *An. gambiae * (*s.s*.) at high temperature was higher in individuals with the 2La inversion than with those without it, presumably due to its association with tolerance to aridity [17]. Gray et al. reported an association of 2La chromosomal inversion with enhanced desiccation resistance of *An. gambiae* [18]*.* The 2La homokaryotypes of *An.gambiae* (*s.s*.) “S” form [now called *An. gambiae* (*s.s*.)] survived longer under desiccation conditions than did heterokaryotypes and individuals with the standard chromosomal conformation [19]. It is also of comparative interest that *An. arabiensis* has the 2La/a conformation fixed, and that species is also more commonly distributed in arid environments compared to *An. gambiae* (*s.s*.) [13].

The differences in tolerance to arid conditions associated with the 2La karyotypes may also be linked to mosquito resting behavior. Individual mosquitoes with the 2La inversion have been found resting indoors where a nocturnal saturation deficit establishes on a diel cycle [15]. In western Kenya, sporozoite infection rates were twice as high in mosquitoes with the 2L+^a^/+^a^ homokaryotype compared to those with the 2La/a inversion homokaryotypes. In a village in Cote d’Ivoire, the frequency of the 2La/a homokaryotype increased following the distribution of insecticide-treated nets [20]. It has been postulated that individuals with the 2La/a homokaryotype may have reduced exposure to insecticides on ITNs or applied on walls due to their relatively more exophilic behavior.

In this study, the frequency of the 2La chromosomal inversion in an *An. gambiae* (*s.s*.) population was investigated in an area of western Kenya that has undergone a marked increase in ownership and use of pyrethroid-treated bed nets over a period of about 15 years [21, 22]. During this time, the *An. gambiae* (*s.l.*) population shifted in the ratio of the two species in the complex that are present in the study area, where *An. arabiensis* became dominant and *An. gambiae* (*s.s*.) became uncommon, reversing the historical trend where *An. gambiae* (*s.s*.) was the dominant species of the pair and countering a prediction from climate change models [23]. In addition, the local *An. gambiae* (*s.s*.) population changed genetically, showing an increase in the L1014S *kdr* allele associated with target site insecticide resistance, from a frequency of 3–100 % (fixed) over a period encompassing about 14 years [22]. The conclusion was that these changes were due to the increased presence of ITNs in the region, which should act more strongly against the relatively more anthropophilic and endophilic *An. gambiae* (*s.s*.) [24] whilst simultaneously selecting for the *kdr* resistance allele [22]. Here, we postulated that the frequency of the 2La chromosomal inversion in the same *An. gambiae* (*s.s*.) population, owing to its association with indoor resting behavior, may change with increased presence of ITNs in the indoor environment.

## Results

### Temporal decline in the frequency of the 2La inversion in *An. gambiae* (*s.s*.)

**Fig. 1:**
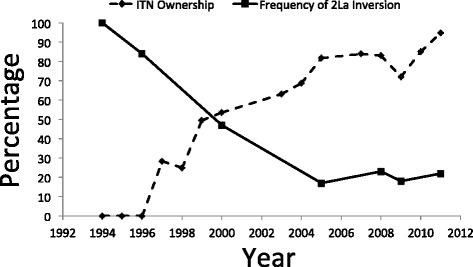
Decline in frequency of the 2La chromosomal inversion in the *Anopheles gambiae* (*s.s*.) population in Asembo, western Kenya, from 1994 to 2011, expressed as a percentage of the total number of chromosomes tested. Household ownership of insecticide-treated bed nets over the same period, expressed as percentage of houses with such nets, is also shown

Four hundred and four *An. gambiae* (*s.s*.) females were analyzed for the 2La/a and 2L+^a^/+^a^ karyotypes (Table 1). At least 46 mosquitoes were analyzed for each year except 2009 when only 17 *An. gambiae* (*s.s.*) were available for testing. The frequency of the 2La chromosomal inversion decreased from 100 % of all chromosomes in 1994 to 17.6 % in 2009 and remained low in 2011 (21.8 %) (Fig. 1). There was a statistically significant, negative correlation between the percentage of chromosomes that had the 2La inversion in the *An. gambiae* (*s.s*.) population and the year that the samples were taken (OR = 0.82, 95 % CI = 0.79–0.85, *P* < 0.001). During the same time interval, the percentage of houses with insecticide-treated bed nets rose from 0 % in 1994 to 95 % in 2011 (Fig. 1). There was a statistically significant, negative correlation between the percentage of chromosomes that had the 2La inversion in the *An. gambiae* (*s.s*.) population and the percentage of houses with insecticide-treated nets (OR = 0.96, 95 % CI = 0.95–0.97, *P* < 0.001).

**Table 1:** Percentage of *Anopheles gambiae* (*s.s*.) females from the Asembo study area of western Kenya with the 2La/a, 2La/+^a^ or and 2L+^a^/+^a^ karyotypes

Year	*n*	2La/a	2La/+^a^	2L+^a^/+^a^	*F*
1994	45	100 (92.3–100)	0 (0.0–7.7)	0 (0.0–7.7)	nd
1996	28	78.6 (63.3–93.8)	3.6 (0.0–10.5)	17.9 (3.6–32.1)	0.86***
2000	50	44.0 (30.2–57.9)	10.0 (1.7–18.3)	46.0 (32.1–59.9)	0.21 ns
2005	83	8.4 (2.4–14.4)	19.3 (10.8–17.8)	72.3 (62.6–82.0)	0.39*
2008	70	12.9 (5.0–20.7)	21.4 (11.8–31.1)	65.7 (54.6–76.9)	0.48*
2009	17	0.0 (0.0–19.5)	41.2 (17.7–64.7)	58.8 (35.3–82.3)	0.61**
2011	151	9.9 (5.1–14.7)	23.8 (17.0–30.7)	66.2 (58.7–73.8)	0.81***

A test of Hardy-Weinberg equilibrium is indicated by the F-statistic

*n* number of female mosquitoes analyzed, *ns* not significant, *nd* not done

*P < 0.05, **P < 0.01, ***P < 0.001

### Hardy-Weinberg equilibrium

Departure from Hardy-Weinberg equilibrium (HWE) was tested for each year of sampling using Wright’s *F* statistic implemented in GENEPOP 4.0 following Petrarca et al. [25] after Brown [26]. Deviation from HW equilibrium occurred in the populations sampled in 1996, 2005, 2008, 2009 and 2011; but not in 2000 (Table 1). The data for 1994 were not tested for Hardy-Weinberg equilibrium, because the frequency of the 2La/a homokaryotype was 100 % in the 46 individuals tested and therefore frequencies of two of the karyotypes had expected values of zero.

### 2La inversion, biting collections and malaria infection

A total of 242 *An. gambiae* (*s.s*.) females was collected in indoor (150) and outdoor (92) biting collections in Asembo in 2011, of which 151 (87 indoor, 64 outdoor) were subjected to PCR for the 2La karyotypes, giving a sample of 302 chromosomes. Of these, 66 (9.9 %) were in the 2La/a inversion and 200 (66.2 %) were 2L+^a^/+^a^. Among the indoor collected mosquitoes, 63.2 % were of the 2L+^a^/+^a^ homokaryotype while 24.1 % had the heterokaryotype (2La/+^a^) arrangement. The corresponding percentages for the outdoor collected mosquitoes were 70.3 and 23.4 %, respectively (Table 2). There was no relationship between karyotype and the location of biting (*χ*^2^ = 1.81, *df* = 2, *P* > 0.05). Fifteen of 148 (10.1 %) of the mosquitoes were positive for *P. falciparum* sporozoites. There was no significant relationship between karyotype and infection rate for sporozoites (*χ*^2^ = 0.71, *df* = 2, *P* > 0.05).

**Table 2:** Percentage of *Anopheles gambiae* (*s.s*.) females collected by HLC from the Asembo study area of western Kenya with the 2La/a, 2La/+^a^ or and 2L+^a^/+^a^ karyotypes by location of feeding

		*n*	%	Lower CI	Upper CI
Indoor	2La	11	12.6	5.6	19.7
	2La+	55	63.2	53.0	73.5
	2La/a+	21	24.1	15.0	33.2
Outdoor	2La	4	6.3	0.3	12.2
	2La+	45	70.3	59.0	81.6
	2La/a+	15	23.4	12.9	33.9

### Rainfall and temperature

**Fig. 2:**
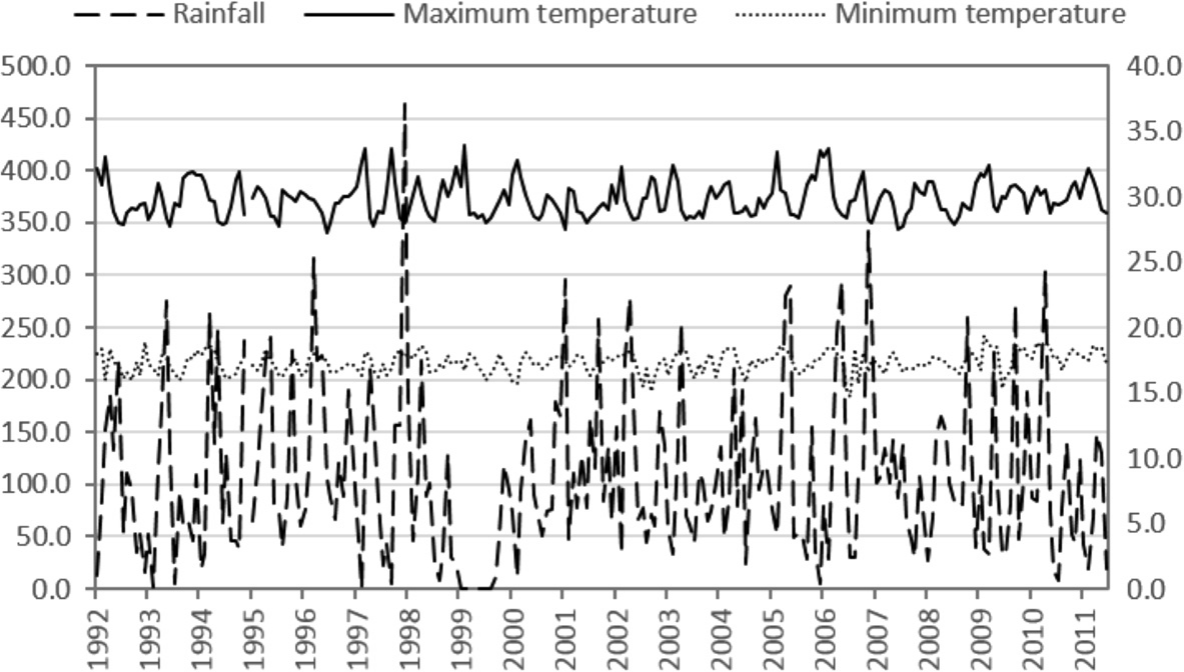
Monthly cumulative rainfall, monthly maximum temperature and monthly minimum temperature in Western Kenya for the years 1994–2011

Monthly cumulative precipitation, and monthly minimum and maximum temperature measured from 1994 to the first six months of 2011 (representing the time interval when mosquitoes were sampled) showed no large upward or downward trends during the time interval when mosquitoes were sampled (Fig. 2). Regression of monthly precipitation and monthly maximum temperature on time yielded correlation coefficients that were not significantly different from zero (precipitation: *r* = -0.008, *P* = 0.901; maximum temperature: *r* = 0.064, *P* = 0.328; *df* = 231 for both analyses). However, regression of monthly minimum temperature (Y) on time (X) showed a gradual and slight warming trend (Y = 0.003X + 17.0, *r* = 0.206, *P* = 0.002, *df* = 231), i.e. minimum monthly temperature warmed 0.04 °C during this 2010-month period.

## Discussion

This study investigated changes in the frequency of the 2La chromosomal inversion over time in populations of *An. gambiae* (*s.s*.) in western Kenya. The decline in the frequency of the 2La chromosomal inversion coincided with increased ITN ownership over a 17.5 year period. The association between household ITN ownership and the frequency of the 2La inversion suggests that ITNs may exert selection on one or more genes that are linked to this inversion. However, further evidence from other sites is necessary to confirm this finding and a clear phenotype which is under selection and linked to the inversion has yet to be identified.

The change in 2La frequency is not the first change in the population genetic structure observed in concert with the scale of ITNs. Several pronounced and measurable changes have occurred in the structure of the *An. gambiae* (*s.s*.) population in the Asembo Bay region of western Kenya coincident with the increase in ownership and use of insecticide-treated bed nets in human dwellings. Amongst these changes have been a decline in density of indoor resting mosquitoes [27] and in probability of daily survival [24], which were both presumed to be due to direct toxic effects of ITNs on the mosquito population. However, recent studies in the Asembo area found that catches of *An. gambiae* (*s.s*.) using CDC light traps placed in sleeping rooms are high compared to indoor resting collections (E. Walker, unpublished observations) and landing rates of *An. gambiae* (*s.s*.) females in human landing catches were higher indoors than outdoors [28]. Therefore, *An. gambiae* (*s.s*.) females continue to visit the indoor environment to obtain blood meals. Also, the frequency of the L1014S *kdr* allele (associated with target site resistance to the pyrethroid insecticides in ITNs) increased within this same *An. gambiae* (*s.s*.) population; it was nearly zero before the large scale trial of ITNs in the late 1990s, but rose to nearly fixation during the same time interval as the decline in the frequency of the 2La chromosomal inversion observed here [22]. Another change during this time interval was that the *An. gambiae* (*s.s*.) was the dominant species of the *An. gambiae* (*s.l*.) species complex in the study area historically, but as ITN ownership rose, it declined and the sister species *An. arabiensis* increased proportionately in both indoor resting collections and in the larval stage [24]. Taken together, these long-term trends suggest that the impact of ITNs on the *An. gambiae* (*s.l.*) complex has been dramatic and has included both numeric and evolutionary processes. This conclusion is consistent with the decline in the frequency of the 2La temporally (Fig. 1) and with the results of the analysis of departure from Hardy-Weinberg equilibrium (Table 1). The dramatic changes in the frequencies of the *kdr* allele and the 2La inversion, two unlinked markers [20, 22], suggests that *An. gambiae* populations are undergoing dramatic population genetic shifts that are possibly due to selection by the increased coverage of ITNs. Investigations into the phenotypes and underlying genotypes that may be selected for by ITNs are warranted to help understand population level shifts and predict how mosquitoes and other insect pests respond to control measures.

To strengthen the inference that presence of ITNs at high levels of ownership in the rural community under study would have the consequence of selecting against individual *An. gambiae* (*s.s*.) with the 2La homokaryotype, it is necessary to demonstrate that ITNs cause changes in spatial distribution of individuals in the population. Indeed, previous studies conducted in Asembo support these processes. Gimnig et al. [29] observed that densities of *An. gambiae* (*s.s*.) were lower in houses of villages supplied with permethrin-treated ITNs compared to villages without ITNs, and further that a measurable density reduction evident as a spatial effect extended hundreds of meters away from the treatment villages. It was likely due to an area-wide population suppression effect. Because population density was assessed by indoor resting collections and the causal inference was direct effects of toxicity of the ITNs resulting in an inimical indoor environment, a logical conclusion is that individuals tending to enter and rest indoors would be selected against compared to those resting outside. The dilemma that the *An. gambiae* (*s.s*.) population in Asembo and Seme face is that humans are mostly accessible for biting indoors at night because of typical human behavior; we found no evidence for shifts in timing or location of biting that might suggest evolutionary responses of these behaviors [28].

The 2La karyotypes have previously been linked to differences in mosquito behavior. In west Africa, individuals with the 2La inversion homokaryotype were more likely to be collected indoors and these varying degrees of endophily associated with inversion polymorphism led to a non-uniform exposure of different inversion karyotype carriers to insecticides [30]. Similarly, Brooke et al. [20] suggested that individuals with the 2La inversion homokaryotype could in effect avoid insecticide-treated surfaces and materials due to their relatively more exophilic behavior. Our data are inconsistent with these previous reports [23] in that the frequency of the inversion karyotype declined with increasing ITN coverage. Although there has been no continuous comparative sampling of the resting *An. gambiae* (*s.s*.) population indoors and outdoors over the same time interval to measure any changes in resting behavior, female *An. gambiae* (*s.s*.) were as abundant resting in clay pots placed as resting stations out of doors as they were resting indoors in the study area in 2005 [31]. Further, males of this species were much more abundant in these outdoor resting stations than they were indoors, at a time when the frequency of the 2La karyotype had declined (Fig. 1). The relationship between 2La/a and 2L+^a^/+^a^ karyotypes and distribution of resting *An. gambiae* (*s.s*.) adults indoors and outdoors in the western Kenya setting needs more research.

Another behavioral trait that may be selected for is the location of biting by *An. gambiae.* If the 2La/a karyotype is associated with indoor biting as a behavioral phenotype in addition to the indoor resting habit, then a reduction in indoor biting would be expected. However, our study on distribution of indoor and outdoor landing catches of *An. gambiae* (*s.s*.) showed no statistically significant tendency to bite indoors or outdoors relative to the 2La/a homokaryotype. Nor was there any difference in sporozoite infection rate between mosquitoes with the homo- or heterokaryotypes, in contrast with results from Petrarca & Beier in which infection rates in the standard homokaryotype (2L+^a^/+^a^) were at least two times higher than for the inverted homokaryotype (2La/a) [12]. Accordingly, it cannot be inferred that the observed decline in the 2La inversion in the *An. gambiae* (*s.s*.) population has led to any change in malaria risk in the human population. A study on spatial and temporal distribution of biting in western Kenya near our study area revealed substantial outdoor biting by *An. gambiae* (*s.s*.) females before ITNs were available, thus outdoor and indoor distribution of bites is not a recently evolved characteristic of the *An. gambiae* (*s.s*.) population in the study area [32]. At the time this part of the study was conducted, the 2La/a homokaryotype had decreased to 9.9 % of individuals in the mosquito population, reducing the sensitivity of the analysis given the modest sample size due to the low population density. Our results suggest that the 2La karyotype appears not to be a good genetic marker of tendency to bite indoors or outdoors.

The 2La inversion includes numerous genes and it is possible that another phenotype is under selection. Indeed, Brooke et al. reported that the 2La inversion is associated with dieldrin resistance [20]. The *kdr* locus which has also increased in frequency coincident with the scale up of ITNs is on the left arm of chromosome 2 but is reportedly not linked to 2La inversion karyotypes [20, 33] and while the 2La inversion declined in frequency over this time, it was still present at frequencies around 20 % while the *kdr* allele rose to near fixation in *An. gambiae* in western Kenya. However, it is possible that other genes associated with pyrethroid resistance, either within the 2La inversion or closely linked to it may be driving the change in frequencies.

An alternative explanation for the observed decline in the frequency of the 2La chromosomal inversion is climate change. Dry, warm conditions should select for the 2La/a karyotype given the association of that inversion with aridity and protection against desiccation [16–19, 34]. Ecological niche modeling predicted that projected climate changes should favor *An. gambiae* (*s.s*.) over *An. arabiensis* in western Kenya [23], which was not upheld in trends of ratios of the two species [24]. Long-term weather variation (Fig. 2) showed no drying or wetting trends during the study, as monthly values of precipitation did not change in the 17.5 year interval nor did maximum monthly temperature increase, while minimum temperature increased only very modestly and would likely select for, and not against, the 2La inversion. Given these trends, there is no evidence that climate change or weather was a driver of the changes in the frequency of the 2La inversion observed here.

Lastly, it should be noted that there are inconsistencies between the current study and previous work in western Kenya. Although we observed the 2La inversion at fixation in 1994, Petrarca & Beier reported the frequency of the 2La to be 43 % from mosquitoes collected in Miwani, approximately 100 km from the current study site, in 1986 and 1987 [12]. Similarly, Collins et al. reported the 2La frequency to be 45 % in 82 *An. gambiae* collected from the Asembo area, the same location as the current study [35]. A potential explanation is the use of different collection methods. The frequency of the 2La inversion was highest in the years when mosquitoes were collected by bed net traps (> 82 %) compared to those collected by PSCs, which may be explained by behavioral differences that biased the results. Sampling using PSC could have been biased towards endophilic mosquitoes and less sensitive to exophagic and exophilic mosquitoes [36]. Collections by Collins et al. were done by indoor aspiration in the morning, which should sample the same population as PSCs. However, 2La inversion frequencies were intermediate in 2000 (46.8 %) when PSCs were first used before falling to less than 20 % in the remaining years. Furthermore, the frequency was also low in 2011 (21.9 %) when mosquitoes were collected by HLC. Therefore, while biases in collection methods cannot be completely discounted, it seems unlikely that this accounts for the dramatic decline in the 2La/a frequency. Another possible explanation for these discrepancies is the use of different assays for the 2La. The current study used a DNA-based assay reported designed around the inversion breakpoints. It was initially reported to be > 94 % accurate when compared to cytological methods. However, recent studies in Tanzania suggest this assay may not be universally applicable as an additional band with an insertion was observed in the assay in Tanzania [37]. Although this polymorphism was not observed in the current study, it is possible that other polymorphisms exist in nature that may affect the DNA-based assay used in the current study.

## Conclusions

*An. gambiae* (*s.s*.) populations in western Kenya experienced a dramatic decline in the frequency of the 2La inversion. The decline was correlated with a scale-up of ITNs which have been shown to be associated with other changes in the structure of *Anopheles* populations in western Kenya. This study provides additional evidence of selective pressure on endophilic and anthropophilic mosquitoes through insecticide pressure by ITNs implemented indoors, which may result in reduced physiological susceptibility to insecticides on nets and/or behavioral adaptations that may reduce the interaction between mosquitoes and insecticides.

## Methods

**Fig. 3:**
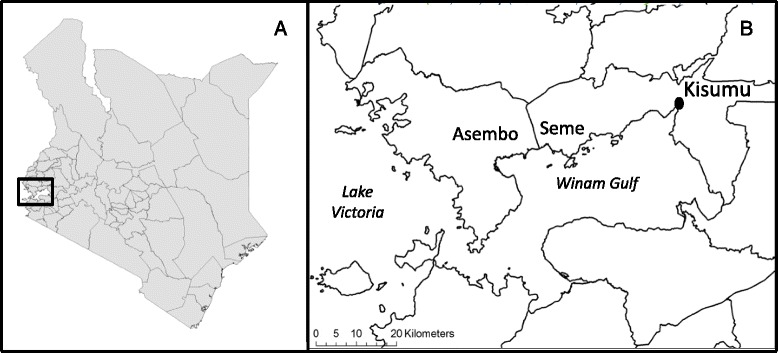
Map of Kenya **a** showing location of sampling area (Asembo and Seme) in western Kenya **b**

The study site consisted of the adjacent communities of Asembo (Rarieda sub-County, Siaya County) and Seme (Seme sub-County, Kisumu County), located *c*. 50 km west of the city of Kisumu in western Kenya [24] (Fig. 3). Presence of efficient malaria vectors and high incidence of *Plasmodium falciparum* infection in the human population combine to establish conditions for holoendemic transmission of *P. falciparum* in this region of the country [38]. The study area is within and immediately adjacent to a region where the effects of permethrin-treated bed nets on vector populations, malaria transmission and malaria infection were analyzed from 1996 to 1999 in a randomized trial [39]. From 1999 through 2003, households in all villages including those originally used as ITN-free controls were provided with permethrin-treated nets which were re-treated every 6 months [27]. From 2003 to 2006, nets were retreated with alphacypermethrin (40 mg/m^2^) at 9–11 month intervals; in 2007, nets were treated with deltamethrin at a dose of 25 mg/m^2^ and the retreatment program was ended [22, 24]. Meanwhile, the Division of Malaria Control (DOMC) of the Ministry of Public Health and Sanitation, Government of Kenya, initiated a program of subsidized ITNs distributed through antenatal and child welfare clinics beginning in 2004 and conducted a mass campaign targeting children under five years in 2006 [21, 22, 24, 40]. In 2011, 11 million LLINs were distributed free of charge in a mass campaign as part of the efforts to achieve universal coverage of all people at risk of malaria. As a result, ownership of insecticide-treated nets increased to high levels in the study area [21, 22, 24].

Mosquito samples used in this study were from indoor resting collections taken from banked specimens that were originally collected by bednet traps (1994 and 1996) or by pyrethrum spray catches (2000, 2005, 2008 and 2009), during the long rains of April–June, described elsewhere [24], with the exception of human landing collections conducted in 2011 (see below). Here we chose samples that were collected within 1 month of each other, to avoid the potential confounding issue of variation in frequency of the 2La inversion in *An. gambiae* (*s.s*.) populations among different seasons [15]. To correlate 2La karyotypes with mosquito feeding behavior, human landing collections were conducted from 12 June to 22 July, 2011, in 75 villages [28]. Collections began at 1700 h with one person collecting inside and one person collecting outside a house, while two collectors rested. At midnight, the collectors switched, allowing the two who collected from 1700 h to rest while the other two collected mosquitoes until 0700 h. Collections were carried out for 45 min during each hour with a 15 min break before resuming collections for the next hour. Collections were done 4 nights per week over the course of 6 weeks.

**Fig. 4:**
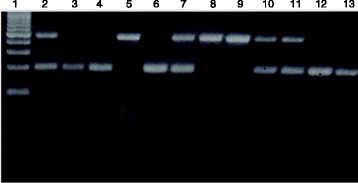
Gel phenotype of the 2La/a and 2L+^a^/+^a^ karyotypes in field collected *Anopheles gambiae* (*s.s*.) from western Kenya, revealed by a PCR-based procedure adapted from White et al. [16]. Lane 1, molecular weight ladder; Lanes 2, 7, 10, 11: 2La/+^a^ heterokaryotypes; Lanes 3, 4, 6, 12, 13; 2L+^a^/+^a^: homokaryotype. Lane 5,8, 9; 2La/a: homokaryotype

Mosquitoes were identified morphologically as *An. gambiae* (*s.l*.), and were dissected into head and thorax, abdomen, legs and wings. DNA was extracted from legs and wings by alcohol precipitation [41] and samples stored at -20 °C. DNA extractions were subjected to PCR amplification of the intergenic spacer region of ribosomal DNA following the method of Scott et al. [42] to identify *An. gambiae* (*s.s*.). Previous studies in east Africa have indicated that only the S form is present in this area [43] and therefore it was assumed that only the S form was present in this study. These same samples were analyzed for presence of the 2La chromosomal arrangement as described by White et al. [16]: primers were designed for amplification of the 2La/a and 2L+^a^/+^a^ proximal breakpoints. The 23A2 and 27A2 primer pairs were used for specific amplification of 492 bp product on the 2La/a breakpoint, while the DPCross5 and 23A2 primers were used for the 2L+^a^/+^a^breakpoint, yielding a 207 bp product. Amplicon size was scored on 3 % agarose gels and compared with a molecular weight ladder (Fig. 4). A single band of 207 bp size was scored as a homokaryotypic standard (2L+^a^/+^a^), whilst a single band of 497 bp size was scored as the homokaryotypic inverted condition (2La/a). Presence of both bands from a single individual was scored as heterokaryotypic (2La/+^a^). All mosquitoes were tested with sporozoite ELISA to determine infection with *Plasmodium falciparum* sporozoites in the salivary glands [44].

### Ownership of insecticide-treated bed nets

The percentage of houses with insecticide-treated bed nets was estimated from household surveys using structured questionnaires [45]. The surveys were conducted annually from 2006. Data from previous years were obtained from surveys conducted at irregular intervals.

### Weather data

Monthly rainfall, maximum temperature and minimum temperature data were obtained from a station at the Kisumu airport maintained by the Kenyan Meteorological Department.

### Statistical analysis

The frequency of the 2La inversion was modeled separately against year and ITN coverage by logistic regression using the GENMOD procedure in SAS (SAS Institute, Cary, NC, USA).

## Abbreviations

2L+^a^/+^a^: standard arrangement on left arm of chromosome 2; 2La/+^a^: heterozygous arrangement on left arm of chromosome 2; 2La/a: inversion arrangement on left arm of chromosome 2; CBRD: Centre for Biotechnology Research and Development; CDC: Centre for disease control and prevention; DNA: deoxyribonucleic acid; ELISA: enzyme linked immunosorbent assay; F: fixation index; F1: first filial; GABA: gamma amino-butyric acid; HLC: human landing Catches; HWE, Hardy-Weinberg equilibrium; IRS: Indoor residual spraying; KEMRI: Kenya Medical Research Institute; LLINs: long-lasting insecticidal nets; PCR: polymerase chain reaction; PSC: pyrethrum spray catches; *s.l.*: *sensu lato*; *s.s*.: *sensu stricto*; WHO: World Health Organization; *X*^*2*^: chi-square
